# Topical delivery of l-ascorbic acid spanlastics for stability enhancement and treatment of UVB induced damaged skin

**DOI:** 10.1080/10717544.2021.1886377

**Published:** 2021-02-23

**Authors:** Mona Elhabak, Samar Ibrahim, Samar M. Abouelatta

**Affiliations:** a Department of Pharmaceutics, Faculty of Pharmacy, Ahram Canadian University, Cairo, Egypt; b Department of Clinical Pharmacy, Faculty of Pharmacy, Ahram Canadian University, Cairo, Egypt

**Keywords:** l-Ascorbic acid, spanlastics, western blot, tape stripping test, photoaging

## Abstract

l-Ascorbic acid (LAA) is considered a powerful antioxidant that protects skin from premature aging. Maintaining the stability of vitamin C remains the biggest challenge in cosmeceuticals. Our main aim is the entrapment of high dose of vitamin C in spanlastic vesicles to provide maximum stability and efficacy. LAA-loaded spanlastics were prepared by ethanol injection method and were characterized for entrapment efficiency (EE%), particles size (PS), polydispersity index (PDI), zeta potential, deformability index (DI) and *in vivo* skin permeation. Selected spanlastics formula composed of span 60 and tween 60 (5:1) showed highest EE% of 89.77 ± 3.61% (w/w), high deformability of 11.13 ± 1.145 as well as good physical and chemical stability for 6 months. Improved drug penetration into stratum corneum (SC) was obtained from spanlastics compared to topical LAA solution. Quantitative real time PCR revealed that MMP2 and MMP9 levels were significantly suppressed in response to LAA spanlastics treated rats by 30.4% and 65.3%, respectively, when compared to the control group after exposure to UV irradiation. Results were confirmed by western blot analysis. Histopathological study of rat skin after UV irradiation revealed that application of LAA-loaded spanlastics provided the highest skin protection compared to UVB and LAA solution treated group which was evident by the normal thick epidermal morphology and the densely arranged dermal collagen fibers. LAA-loaded spanlastics successfully improved LAA stability, skin permeation and antioxidant protection against skin photodamage.

## Introduction

Premature skin photoaging is one of the most common harmful effects of chronic exposure to UV irradiation (Pallela et al. 2010). Vitamin C is a very potent naturally occurring antioxidant drug. Human body cannot synthesize vitamin C due to the absence of the enzyme l-glucono-gamma lactone oxidase. This represents a major challenge toward utilization of vitamin C which suffers severe stability problems as well as difficulty in delivery to the targeted site (Al-Niaimi & Chiang 2017). Being a cofactor in collagen biosynthesis, vitamin C provides its well-known antiaging and anti-wrinkling activity. This is achieved by increasing collagen synthesis, as well as diminishing collagen degradation, hence melanin production and reduction of skin pigmentation (Rattanawiwatpong et al. 2020).

Although oral doses of vitamin C are somehow high, only a small fraction will be biologically available and active in the skin as its absorption in the gut is limited by active transport mechanism which in turn leads to low and erratic bioavailability (Gref et al. 2020). Therefore, topical formulations are considered the best choice in dermatological issues. It was clinically proven that the effective concentration of vitamin C in topical preparations should range from 10 to 20% (w/v). Lower concentrations were ineffective, while higher concentrations might cause certain irritations (Telang 2013, Rattanawiwatpong et al. 2020).

Nowadays several creams and serums are widely marketed in the field of pharmaceuticals and cosmeceuticals. As vitamin C is highly vulnerable to oxidation, in aqueous systems, that threatens shelf-life stability of the final product. This is quite confirmed by the darkening of vitamin C aqueous solutions and emulsions under normal storage conditions (Caritá et al. 2020). This instability represents a great challenge to formulators. Several attempts have been adopted to overcome stability problems of vitamin C such as: monitoring the oxygen levels, working in acidic medium, reducing water content in the formulation as well as esterified derivatives of vitamin C (Caritá et al. 2020). However, it was found that formulations containing derivatives of vitamin C were unable to raise the skin content of l-ascorbic acid (LAA) which is the only biologically active form of Vitamin C (Pinnell et al. 2001).

Spanlastics are classified as ultra-deformable or elastic vesicles (Sallam et al. 2020). Unlike liposomes and niosomes, spanlastics contain surfactants in their composition employed as edge activators for destabilization of the lipid bilayer thus increasing its deformability and enhancing percutaneous absorption of drugs (Doppalapudi et al. 2017).

The objective of the present study was the formulation of LAA as nano vesicular spanlastics to overcome stability problems and enhance drug permeation to achieve best clinical results.

## Materials and methods

### Materials

d-α-Tocopherol polyethylene glycol 1000 succinate (TPGS) was gifted by Isochem (France). LAA was supplied by El-Gomhouria Co. (Egypt). Sorbitan monopalmitate (span 40), sorbitan monostearate (span 60), polyoxyethylene sorbitan monostearate (tween 60), polyoxyethylene sorbitan monooleate (tween 80), polyvinyl alcohol (PVA), methanol, and absolute alcohol (97%) were provided from El-Nasr Pharmaceutical Co. (Egypt). Spectra Por semipermeable membrane tubing (MWCO 12,000–14,000) was obtained from Spectrum Laboratories Inc. (Rancho Dominguez, CA).

### Preparation of LAA-loaded spanlastics

Ethanol injection method was employed to prepare spanlastics dispersions loaded with LAA as previously described by Sallam et al. (2020). In brief, LAA and span were dissolved together in ethanol kept at 50 °C, the solution was then injected slowly into preheated aqueous solution (60 °C) containing an edge activator (EA). The resulting hydro-alcoholic dispersion was kept on the magnetic stirrer (Ika, Germany) under vigorous stirring till complete evaporation of alcohol. The excess entrapped drug was removed by dialysis method (Machado et al. 2018). Briefly, 3 mL of LAA-loaded spanlastic dispersions were placed into a dialysis bag (Sigma Aldrich, cut off two kDa MW), tied at both ends and immersed in 200 mL of water with stirring. At appropriate time intervals, the samples were withdrawn from the receiving medium and analyzed for drug content using suitable UV spectroscopy at 243 nm, until no more drug was detected (Yasam et al. 2014). The prepared formulae were further stored in amber-colored glass containers till further investigation.

Study was done following systemic stepwise approach. First, the effect of the type of different span grades (20, 40 and 80) on loading parameters was investigated. The formula showing promising results was further studied for the effect of EAs type (tween 60, tween 80, and TPGS). Then both the ratio between aqueous phase and organic phase and the ratio between span and EA were varied as displayed in Table 1.

**Table 1.** Composition and Characteristics of LAA-loaded spanlastics.

Formula	Span Type and amount (mg)	EA Type and amount (mg)	Amount of LAA	Organic phase: Aqueous phase	%EE^a^	LC^a^ %	PS^a^ (nm)	PDI^a^	ZP^a^ (mV)	DI^a^
F 1	Span 40 (100 mg)	Tween 60 (100 mg)	20 mg	1:1	NA	NA				
F 2	Span 60 (100 mg)	Tween 60 (100 mg)	20 mg	1:1	42.75 ± 1.56	3.88 ± 0.20				
F 3	Span 80 (100 mg)	Tween 60 (100 mg)	20 mg	1:1	NA	NA				
F 4	Span 60 (100 mg)	TPGS (100 mg)	20 mg	1:1	31.88 ± 0.68	2.89 ± 0.08				
F 5	Span 60 (100 mg)	Tween 60 (100 mg)	20 mg	1:2	35.44 ± 0.92	3.22 ± 0.11				
F 6	Span 60 (100 mg)	Tween 80 (100 mg)	20 mg	1:1	22.29 ± 2.31	2.02 ± 0.29				
F 7	Span 60 (500 mg)	Tween 60 (100 mg)	20 mg	1:1	84.40 ± 4.38	2.72 ± 0.19				
F 8	Span 60 (500 mg)	Tween 60 (200 mg)	20 mg	1:1	64.5 ± 1.91	1.79 ± 0.07				
F 9	Span 60 (500 mg)	Tween 60 (100 mg)	1.25 g	1.4:1	86.79 ± 6.53	61.29 ± 6.52				
F 10	Span 60 (500 mg)	Tween 60 (100 mg)	2.5 g	1.4:1	89.77 ± 3.61	74.31 ± 4.21	642.6 ± 16.54	0.533 ± 0.12	−23.5 ± 1.34	11.13 ± 1.145
F 11	Span 60 (500 mg)	Tween 60 (100 mg)	5g	1.4:1	39.37 ± 0.30	35.66 ± 0.38				

^a^ Data are presented as mean ± SD (*n* = 3).

### *In vitro* characterization of LAA-loaded spanlastics

### Determination of percentage entrapment efficiency (%EE) and percentage loading capacity (LC%)

The percent drug entrapped was evaluated for all the prepared formulae. Briefly, 1 ml of the dialyzed samples was further sonicated (Elmasonic S 300, 220–240 V) with 10 ml warm ethanol for 15 min to ensure complete vesicles degradation (Tayel et al. 2015). LAA was determined using UV-vis spectrophotometer (JASCO, Tokyo, Japan) at 243 nm. The percent of drug entrapped, and drug loaded were calculated according to the following equations:

(1) % EE=amount of LAA in vesicles/total amount of LAA*100

(Talebi et al. 2019).

(2) %LC=amount of LAA in vesicles/total weight of LAA*100

The formula showing highest %EE and %LC was selected for further characterizations.

### Determination of particle size (PS), polydispersity index (PDI) and zeta potential

Particle size and size distribution of the selected formula were evaluated using zetasizer (Nano ZS, Malvern, UK) employing dynamic light scattering technique (DLS). Brownian motion of the particles results in fluctuations in light scattering that are further analyzed by DLS technique giving the estimated PS (Hassan et al. 2015). Zeta potential of the chosen formula was analyzed using doppler electrophoresis laser velocimetry using Zetasizer (Nano ZS, Malvern, UK).

### Morphology of the vesicles through transmission electron microscope (TEM)

The morphology and the size of the selected formula were further analyzed using TEM (Jeol JEM 1230, Tokyo, 177 Japan). Briefly, one drop of selected formula was placed on the copper grid and left till completely adsorbed for approximately 30 min. The dried sample was then visualized using TEM at 80 kV (Farghaly et al. 2017).

### Elasticity determination

Elasticity of the selected formula (F10) was evaluated by adopting extrusion method. One gram of the prepared colloidal dispersion was extruded through 0.2 µm pore size nylon filter for 3 min under constant pressure of 2.5 bar (Haug Kompressoren AG; Büchi Labortechnik AG, Flawil, Switzerland). The following formula was used for calculating the deformability index (DI):

$$\text{DI}\;=\text{ j }{\left(\text{rv}/\text{rp}\right)}^{2}$$

where j: the weight of the sample (g) extruded.

*r _v_*_:_ the size of the spanlastics after extrusion (nm).

*r_p_*_:_ the pore size of the nylon filter (nm) (Aziz et al. 2019).

### Fourier transform-infrared spectroscopy (FT-IR)

The selected formula (F10) was composed of LAA, span 60, and tween 60. FT-IR studies were carried out to investigate chemical compatibility between these three components. Physical mixtures as well as the selected formula (F10) were also investigated. Solid samples were mixed and compressed with KBR to form compact disks, whereas liquid samples [Tween 60, and selected formula (F 10)] were directly determined. Both solid and liquid samples were scanned from 4000–400 cm^−1^ in transmission mode using FT-IR spectrophotometer (IRspirit, Schimadzu, Japan).

### Effect of storage conditions on physical and chemical properties of spanlastics

The physical and chemical stability of the selected formula (F10) was investigated at room temperature (25 °C) for 6 months. Spanlastics dispersion was stored in sealed amber colored glass vials and stored. Samples were withdrawn at different time intervals (1, 2, 3 and 6 months) to detect any visual changes; %EE, PS, PDI, and zeta potential were also evaluated for any changes.

### *In vivo* skin permeation [tape stripping (TS) test]

The selected formula (F10) was compared to LAA topical solution (10%w/v) for skin permeation using tape stripping test. LAA topical solution was prepared by dissolving 10 g LAA in 100 mL deionized water. Six healthy female volunteers with normal skin condition were chosen for this study. The protocol was approved by the faculty of pharmacy, Ahram Canadian University Ethics Committee (PT 2/2020). Volunteers were randomly assigned to each of the two groups. Four application sites (5 cm in diameter) were determined on each forearm of each subject representing different application times (0.25, 0.5, 1, and 2 hours). Sites were apart from each other to avoid overdosing and were also away from arm and wrist. Two hundred and fifty microliters of each formula were applied once at each site. At the end of each duration, the excess formula (unabsorbed) was removed using cotton. For quantification of drug penetrated stratum corneum (sc), the tape was pressed forward and backward to stretch the skin surface to ensure neglectable effect of furrows and wrinkles on TS test. The tapes were removed rapidly at an angle of 45° (Abdel-Salam et al. 2017). Tape stripping at each site was carried out using fifteen adhesive tape strips (ScotchTM tape: No. 845 Book Tape 3 M Co., MN) to ensure complete removal of sc. The cotton of each site (unabsorbed drug) was individually placed in an analysis bottle with 10 ml ethanol, shaken for 5 min, then sonicated for 30 min to ensure complete extraction of the drug. The 15 adhesive strips of each site for each volunteer were collected in assay bottles and the same steps previously described for drug extraction were followed. Ethanolic extracts for each application time were then collected, filtered through Millipore filter 0.2 µm and were further analyzed spectrophotometrically at 243 nm for quantification of LAA (Gonçalez et al. 2013). The percent of penetrated drug was plotted against application time for both formulae.

### *In vivo* study

Twenty-four Swiss albino male rats weighing 170–200 g were supplied by the animal house of Ahram Canadian University, Egypt. The experimental procedures were performed as directed by the principles of laboratory animal use and care and approved by Faculty of Pharmacy, Ahram Canadian University Ethics Committee (PT 2/2020). Animals were stabilized one week before study in standard environmental conditions. The animals had free access to commercial chow and tap water *ad libitum*.

The rats’ dorsal skin was shaved by an electric clipper. Rats were randomly divided to four groups (six rats each). Group I: control*-*group. Group II: UVB treated group. Group III: animals treated with UVB then LAA solution (10%w/v). Group IV: animals treated with UVB and LAA-loaded spanlastics (F10). Treatments were applied topically to the dorsal areas of rats’ skin 30 min before each UVB exposure. Rats’ dorsal regions were exposed to UVB irradiation (15 W 254 nm, 100 mW/cm^2^) 5 min every day for about 3 months. Twenty-four hours after last treatment rats were euthanized by cervical dislocation then fragments of skin were excised from the dorsal area of each rat to be used for investigations.

### RNA isolation and RT-PCR

Total RNA was isolated from skin fragments by pure script isolation kit (Genera systems, Inc., Minneapolis, MN, USA). RNA was reverse-transcribed into cDNA. GoTaq PCR master mix was used to perform qPCR (Promega Co., Madison, WI, USA). The standard protocol: 95 °C for 10 min, then 40 cycles of 95 °C for 15 s, followed by 60 °C for 1 min then 60 °C for 30 s was visualized on a 7500 Real-Time PCR System (Applied Biosystems, Foster City, CA, USA). The primer sequenceofMMP-2 was as follow 5′-ACCGTCGCCCATCATCAA-3′ (forward), 5′-CCTTCAGCACAAAGAGGTTGC-3′ (reverse); and for MMP-9 was 5′-TGTCCAGACCAAGGGTACAGC-3′ (forward), 5′-GAAGAATGATCTAAGCCCAGCG-3′ (reverse); and 5′-ATGCTCTCCCTCACGCCATC-3′ (sense) and 5′-CAGGATTCCATACCCAAGA-3′ (antisense) for β-actin used as an internal control (Ezzat et al. 2016). Relative quantifications were calculated using the 2^−ΔΔCt^ method.

### Western blot

For homogenized skin tissues, protein levels were quantified using extraction kit supplied by Bio-Rad Inc. Equal protein amount from each sample was resolved on SDS-10%-PAGE. After electrophoresis, the proteins were transferred to Immunoblot™ polyvinylidene difluoride membranes (GE10600021 Sigma, Sigma-Aldrich, MO, USA), then the membrane was blocked with tris-buffered saline with Tween 20 buffer and 3% bovine serum albumin for 1 hour at room temperature. Primary antibodies (MMP2, MMP9) were diluted in TBST and Incubated overnight in each primary antibody solution (with dilution 1:500). Incubation was done in the HRP-conjugated secondary antibody (Goat anti-rabbit IgG-HRP-1 mg Goat mab, Novus Biologicals, Littleton, CO) for 1 hour at room temperature. The chemiluminescent detection was implemented using a CCD camera-based imager. Results of the target proteins were expressed against control sample beta actin (housekeeping protein) by protein normalization on the ChemiDoc MP imager (Afaq et al. 2009).

### Histological study

Skin fragments from different groups were fixed in 10% neutral buffered formalin then cleared in xylene and embedded in paraffin. The sections were cut at 4 µm thickness. The obtained sections were deparaffinized and stained by hematoxylin & eosin stain for examination (Bancroft & Gamble 1996).

#### Statistical analysis

All formulations were prepared and reported in triplicates. Results are expressed as mean ± standard deviation. The statistical significance of difference between the prepared formulae was evaluated by one-way ANOVA and Tukey’s post hoc test using graph pad prism. Differences between the two formulae in the tape stripping test were compared using Student’s *t*-test. For quantitative mRNA analysis, mean (*n* = 6/group) and standard deviation of the mean were calculated. Comparison was carried out using one-way ANOVA followed by the Tukey-Kramer multiple comparison test using Instat software. A probability level of ≤.05 was accepted for significance in all statistical tests.

## Results and discussion

### Preparation of LAA-loaded spanlastics

Spanlastic vesicles were successfully prepared by the ethanol injection method using only span 60 as surfactant and tween 60, tween 80 and TPGS as EAs. Vesicles prepared with Span 80 and Span 40 showed precipitation and failed to form spanlastic vesicles. Similar results were reported in previous studies using span 80 and span 40, as they exhibited a high degree of disruption, aggregation, and instability. While span 60 is composed of lipophilic saturated alkyl chains that impart more sustainability to the spanlastics (Kakkar & Kaur 2011)**.**

### Determination of entrapment efficiency (EE%) and loading capacity (LC)

LAA was successfully entrapped in all spanlastic vesicles prepared with Span 60 as shown in Table 1. The use of tween 60 as EA in F2 provides significant increase in %EE [42.75 ± 1.56%(w/w)] compared to F4 [31.88 ± 0.68%(w/w)] and F6 [22.29 ± 2.31%(w/w)] using TPGS and tween 80, respectively. Varying the ratio of organic phase to aqueous phase from 1:1 (F2) to 1:2 (F5) shows significant decrease in %EE from 42.75 ± 1.56% (w/w) to 35.44 ± 0.92% (w/w), respectively. F2 showing highest %EE [42.75 ± 1.56% (w/w)] and %LC [3.22 ± 0.11% (w/w)] was selected for further study. Increasing the amount of span 60 to 500 mg (F7) was accompanied with significant increase in EE% [84.40 ± 4.38%(w/w)]. On the other side, doubling the amount of EA (F8) results in significant decrease in EE% [64.57 ± 1.91% (w/w)].

As LAA was reported to be effective as anti-aging in topical preparations from 5 to 10%(w/v), so increasing loading capacity was one of our main objectives (Kakkar & Kaur 2011). F7 showing best %EE was chosen for increasing drug loading. Increasing the theoretical drug loading from 100 mg (F7) to 1.25 g (F9) and 2.5 g (F10) caused no significant effect (*p* > .05) on %EE. Conversely, further increase in loading amount to 5 g (F11) was accompanied with significant decrease (*p* ˂.05) in EE% to 39.37 ± 0.30% (w/w). The decrease in EE% might be due to the fixed amount of surfactant available for drug entrapment. Formula F10 showed the highest %EE [89.77 ± 3.61% (w/w)] and LC% [74.31 ± 4.21% (w/w)] and hence was selected for further characterization.

It was obvious that the EA type had a significant effect on %EE (*p* ˂.05). Tween 60 was showing higher EE% followed by TPGS and then Tween 80. The effect of EA type on the drug EE% was previously stated in literature. These results were in accordance with those obtained by Hao et al. and Tayel et al. where higher %EE of the hydrophilic drugs, colchicine and pravastatin sodium were attained using polysorbate 60 compared to 80. The saturated alkyl chain of Tween 60 would enhance the rigidity of vesicular membrane and prevent drug escape. While the possibility of bending of the unsaturated alkyl chain of Tween 80 would affect the tightness of the vesicular membranes and facilitate drug leakage into the aqueous media (Hao et al. 2002; Tayel et al. 2015). This might explain the better %EE obtained when using the saturated chain of TPGS compared to the unsaturated Tween 80. Goindi et al. and Elsherif et al. reported the increase in %EE using sodium deoxycholate instead of tween 80 in the preparation of cetirizine dihydrochloride and terbinafine hydrochloride nanovesicles, respectively. Researchers suggested that Tween 80 could enhance micellar solubilization of the active ingredient in water (Goindi et al. 2013; Elsherif et al. 2017). Another study had attributed the low %EE of Tween 80 versus cremophor when used as EA in the preparation of nanospanlastics to the steric branched structure of Tween 80 which prevented drug entrapment (Badria et al. 2020). The role of EA on EE% was highlighted by Tayel et al. The %EE of the hydrophilic drug pravastatin sodium significantly decrease when the EA was removed from nanospanlastics. This might be explained by the formation of hydrogen bond between the polar head of tween and the hydroxyl group of pravastatin sodium (Tayel et al. 2015).

### *In vitro* characterization of the selected formula (F10)

#### PS, PDI, and zeta potential measurements

PS and PDI determined by DLS were 642.6 ± 16.54 nm and 0.533 ± 0.12, respectively. A PDI below 0.7 indicates that the sizes of the prepared vesicles are almost homogenous (Danaei et al. 2018; Fahmy et al. 2018). In order to achieve the effective concentration of LAA in the topical formulation [10% (w/w)], large amount of span 60 was incorporated which resulted in increasing the size of the vesicles. Along with the high drug loading led to the expansion of the vesicle and the increase in the median diameter (El-Mahdy et al. 2020). The approximate size of vesicles previously reported was 0.5 to 1.0-µm diameter (Kakkar & Kaur 2011). Zeta potential of the selected formula (F10) was found to be −23.5 ± 1.34 mV. The obtained negative zeta potential was previously reported in spanlastics prepared with nonionic surfactants (Aziz et al. 2018). Although the absence of charge inducing agents, the negative surface charge obtained was demonstrated to be the result of the adsorption of the hydroxyl counterions on the surface of nanovesicles (Essa 2010). Pawar et al. (2016) explained that the ionization of free groups present on the niosomal surface was responsible for the negative zeta of bifonazole-loaded niosomes prepared with span 60.

#### Transmission electron microscopy (TEM)

**Figure 1.**
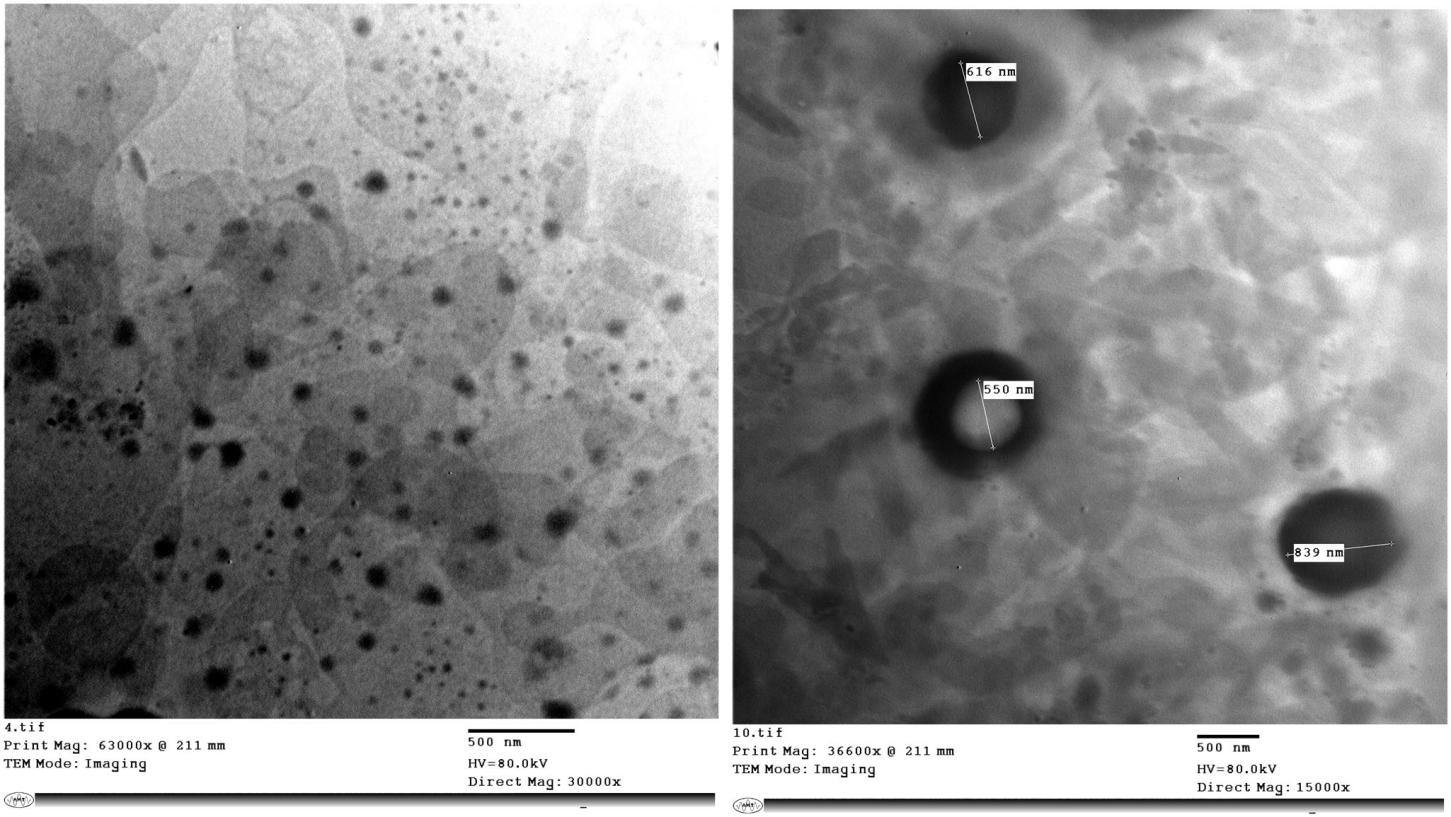
TEM micrograph of the optimized formula (F10).

Figure 1 reveals that the nanovesicles of the selected formula F10, were spherical, uniform in shape with no aggregation and their sizes were in agreement with the results obtained by Zetasizer.

#### FT-IR analysis

**Figure 2.**
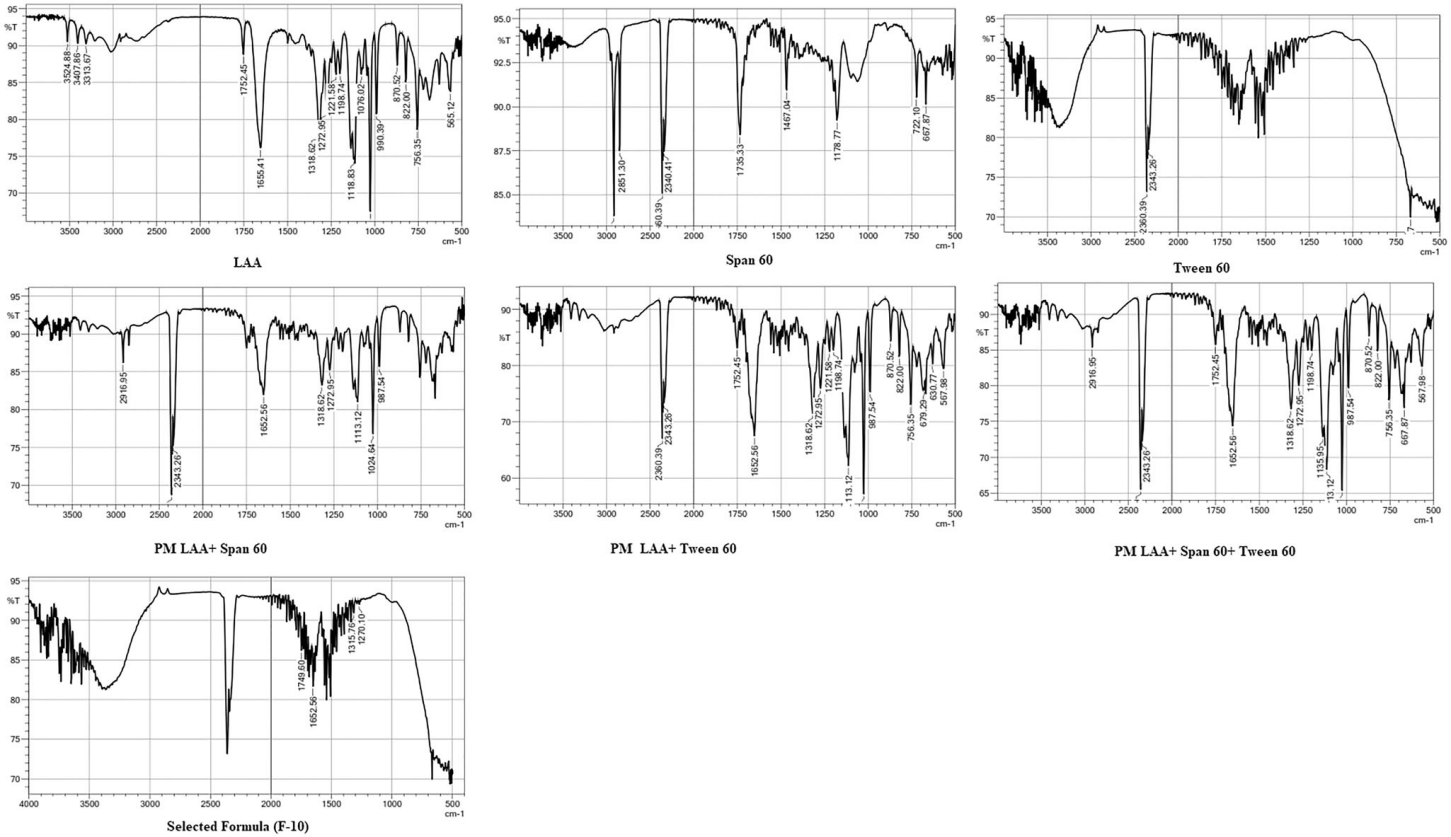
FTIR spectrum of LAA, Span 60, Tween 60, physical mixture (PM) of LAA and span 60, PM of LAA and Tween 60, PM of LAA, span 60 and tween 60, optimized formula (F10).

The FTIR spectra of pure drug, pure surfactant (span 60), pure EA (tween 60), their physical mixture and final selected formula are shown in Figure 2. FT-IR spectrum of lactone C = 0, the stretching vibration of C–C double bond and the peak of enol-hydroxyl were observed at 1750 cm^−1^, 1674 cm^−1^ and 1318 cm^−1^, respectively (Lohmann et al. 1984). The main absorption band of the characteristic peaks of pure LAA did not disappear in spanlastics formulation indicating no drug excipient interaction between LAA and the other excipients.

#### Elasticity

Elasticity of spanlastics was assessed by deformability index (DI) using extrusion method as previously described. DI gives indication of the ability of vesicles to squeeze through the skin pores which are smaller in size than the prepared nanovesicles. DI of the selected formula (F10) was 11.13 ± 1.145 indicating good elasticity and high deformability. The presence of EA in spanlastics is responsible for the destabilization of the lipid bilayer improving its elasticity (Hussain et al. 2017). The non-bulky hydrocarbon chains of tween along with its unsaturated alkyl chain render it more deformable, elastic, and hence more membrane permeable. Span 60, the main component of spanlastics had a membrane softening effect as previously reported (Gupta et al. 2005).

### Effect of storage conditions on physical and chemical properties of spanlastics

No change in color was reported after 6 months. Mean and standard deviation comparison was carried out using one-way ANOVA followed by the Post-hoc test using graph pad software. Spanlastics did not show any significant change (*p* > .05) in PS, PDI or zeta potential excluding aggregation (Table 2). Furthermore, no significant change (*p* > .05) in %EE was determined confirming LAA stability. Vitamin C in topical formulations undergoes photooxidation as well as chemical oxidation in dark. The entrapment of LAA inside the hydrophilic core of nanovesicles protected the vitamin from surrounding aqueous media and hence prevented its oxidation and subsequent degradation. Farahmand *et al.*, showed the highest protection of LAA when formulated in W/O/W multiple emulsions. It also demonstrated that the formation of mixed micelles and the entrapment of LAA in the hydrophilic head of surfactant might have a crucial role for LAA stability (Farahmand et al. 2006).

**Table 2.** Effect of storage on LAA-loaded spanlastics.

Time (month)	PS (nm)^a^	PDI^a^	Zeta potential^a^	%EE^a^
0	642.6 ± 16.54	0.533 ± 0.12	−23.5 ± 1.34	89.7 ± 3.61
1	633.4 ± 5.32	0.439 ± 0.11	−20.1 ± 2.41	90.2 ± 4.03
2	645.7 ± 9.64	0.486 ± 0.14	−25.5 ± 1.04	89.1 ± 2.72
3	641.8 ± 11.87	0.590 ± 0.12	−22.1 ± 1.92	88.8 ± 3.37
6	639.9 ± 0.39	0.613 ± 0.11	−20.6 ± 2.56	89.5 ± 0.87

^a^Data are presented as mean ± SD (*n* = 3).

*Statistically significant different (*p* ≤ 0.05).

### *In vivo* skin permeation study

**Figure 3.**
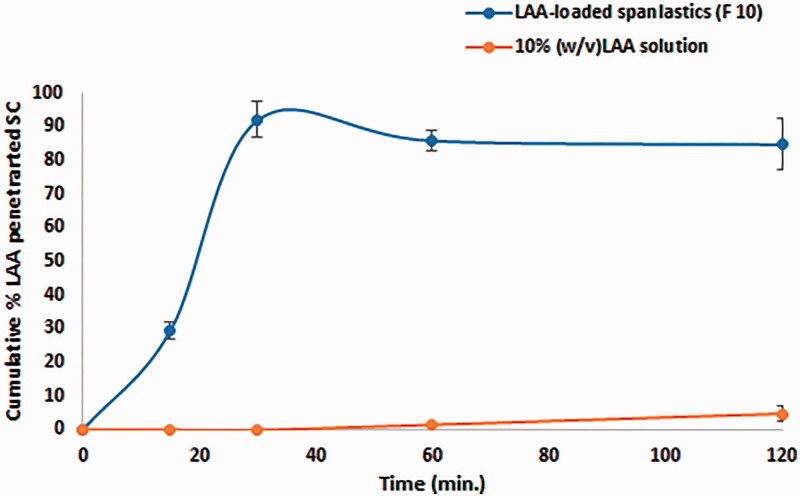
Cumulative percent of LAA absorbed through human SC.

Tape stripping technique was applied to investigate *in vivo* skin permeation. It is a minimally invasive technique to evaluate drug penetration through the skin, and to quantify the amount of drug accumulated in the stratum corneum (SC) (Sanna et al. 2007). Figure 3 shows the percent of the cumulative amount of LAA penetrating the skin from both LAA solution and LAA-loaded spanlastics after different application times. The percent of drug retained into SC after application of spanlastics were found to be 29.44 ± 2.67%(w/w) after 0.25 h and reached its maximum 92.03 ± 5.32%(w/w) after 0.5 h. On the contrary, the drug solution showed negligible penetration of only 7.51 ± 3.16%w/w in the SC after 2 h of application. Being hydrophilic and charged molecule (pKa 4.2), LAA suffers from poor skin penetration (Al-Niaimi & Chiang 2017). Hence, the pH of LAA formulations should be adjusted below 3.5 to ensure skin penetration (Pinnell et al. 2001). Although the measured pH for LAA-loaded nanospanlastics (F10) and LAA solution were 4.55 and 2.38, respectively, nanospanlastics showed higher skin concentration. This might be attributed to the enhanced elasticity and skin penetration ability of the spanlastics nanovesicles which enable them to successfully deliver their payload inside SC. Hence, LAA when incorporated into spanlastics, a significant increase in SC penetration was achieved within only two hours.

### The expression of MMP2, MMP9 genes in rats’ skin

Quantitative real time PCR showed that UV irradiation significantly elevated levels of MMP2 and MMP9 by 8.2- and 1.8-fold, respectively (*p* < .001), compared to untreated control group. There was no significant change arose in MMP2 and MMP9 levels in LAA solution treated rats compared to UVB control group. The induction of MMP2 and MMP9 was significantly downregulated in response to LAA spanlastics (F10) by 30.4% (w/w) and 65.3%(w/w), respectively, compared to UVB control group (Table 3). These results were in accordance with Kim et al. (2011) which reported that exposure to UV irradiation causes the upregulation of different MMPs that break dermal matrix protein such as collagen and elastin of the extracellular matrix. Vicentini et al. (2011) previously proved that the inflammatory cytokines induced MMP-9 expression through NF-KB in keratinocytes exposed to UVB.

**Table 3.** The expression of MMP2 and MMP9 genes in rats’ skin.

	Group I	Group II	Group III	Group IV
	Control group^a^	UVB control^a^	LAA solution treated group^a^	LAA spanlastics (F 10) treated group^a^
MMP2	0.333 ± 0.01	2.738 ± 0.04	2.595 ± 0.157	0.834 ± 0.04*
MMP 9	2.223 ± 0.03	4.029 ± 0.04	3.857 ± 0.06	2.633 ± 0.04*

^a^Data are presented as mean ± SD (*n* = 3).

*Statistically significant difference compared to group II (*p* ≤ 0.05).

### Western blot analysis

**Figure 4.**
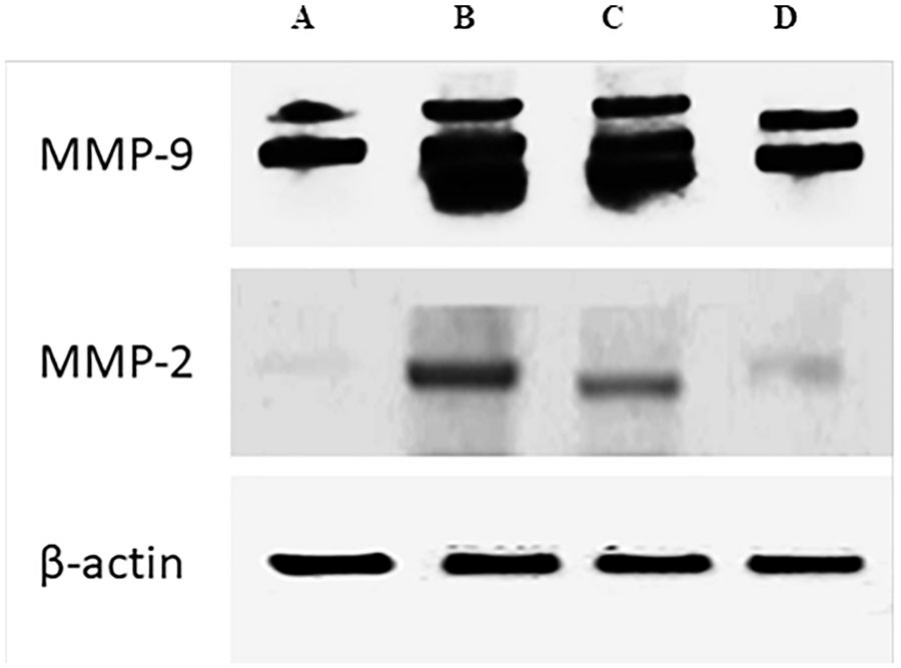
Western blot analysis: (A) protein expression in untreated control group; (B) protein expression in UVB-treated group; (C) protein expression in UVB and LAA solution treated group; (D) protein expression in UVB and LAA-loaded spanlastics treated group in skin biopsies.

Skin biopsies were obtained and subjected to western blotting for detecting MMP-2 and MMP-9. Results showed that UVB irradiation dramatically elevated MMP2 and MMP9 protein expression. Similarly, LAA solution treated rats showed increased proteins expression. However, topical treatment with LAA spanlastics (F10) considerably suppress MMP2 and MMP9 protein expression as represented in Figure 4 confirming results obtained by quantitative real time PCR.

### Histopathological study

**Figure 5.**
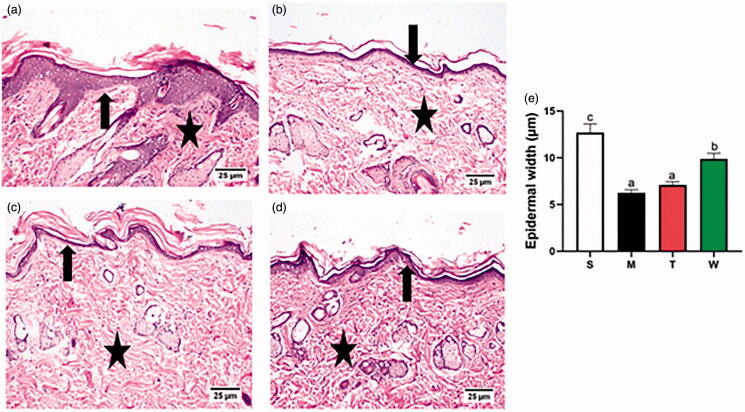
Photomicrographs of H&E stained paraffin sections of skin tissues of (a) untreated control group showing normal histology of epidermis and dermis (arrow) and tightly arranged collagen fibers (star). (b) UVB treated group showing thin epidermis thickness admixed with atrophy of skin adnexa (arrow) and loosely arranged dermal collagen fibers (star). (c) UVB and LAA solution treated group showing thin epidermal layer (arrow) and intact dense dermal collagen bundles (star). (d) UVB and LAA-loaded spanlastics treated group showing normal epidermal morphology admixed with regular arrangement of epidermal cell layers and well-integral structures of the dermis (arrow) and densely arranged collagen fibers (star). (e) Epidermal width measurements in different groups, values are presented as means ± Stdev, a, b and c indicate significant difference at *p* ˂ .05. S: Untreated control M: UVB treated group T: UVB and LAA solution treated group W: UVB and LAA-loaded spanlastics treated group.

Skin of untreated control group showed no histological alteration in the structure of the epidermis that is composed of stratified squamous keratinized epithelium rested on intact basement membrane with healthy underneath dermal layer containing hair follicles and associated skin adnexa (Figure 5(a)). Animals from UVB treated group exhibited atrophied and loosely arranged epidermis with marked decrease in the epidermal thickness accompanied by disorganized dermal collagen fibers with atrophy of the associated dermal adnexa (Figure 5(b)). The examination of UVB and LAA solution treated group showed thinning of epidermal layer associated with apparently normal dermal collagen bundles that appeared densely arranged and interlacing into a network (Figure 5(c)). Administration of LAA-loaded spanlastics resulted in marked improvement of histological abnormalities which was evidence by apparently normal thick epidermal morphology and densely arranged dermal collagen fibers that revealed neatly and interwoven network bundles (Figure 5(d)). The epidermal thickness analysis showed a significant decrease in UVB treated group and LAA solution treated group in comparison with untreated control group. The LAA-loaded spanlastics treated group achieved the highest protection compared to UVB and LAA solution treated group (Figure 5(e)).

## Conclusions

In this study, LAA-loaded spanlastics were successfully prepared using ethanol injection method. The method was optimized to achieve elastic highly permeated nanovesicles with high EE% [89.77 ± 3.61% (w/w)] encapsulating effective topical dose of LAA as an antiaging drug. Quantitative real time PCR, western blot analysis, and histopathological studies showed that LAA-loaded spanlastics had the most effective and well-tolerated treatment compared to LAA solution. Finally, LAA-loaded spanlastics provided clinical improvement of the UV induced damaged skin and ultrastructure, suggesting that elastic nanovesicles had a synergistic effect on collagen synthesis and melanin formation on photoaged skin.
